# An L_p_ (0 ≤ *p* ≤ 1)-norm regularized image reconstruction scheme for breast DOT with non-negative-constraint

**DOI:** 10.1186/s12938-017-0318-y

**Published:** 2017-03-03

**Authors:** Bingyuan Wang, Wenbo Wan, Yihan Wang, Wenjuan Ma, Limin Zhang, Jiao Li, Zhongxing Zhou, Huijuan Zhao, Feng Gao

**Affiliations:** 1 College of Precision Instruments and Optoelectronics Engineering, Tianjin University, Tianjin, 300072 China; 2 Cancer Institute and Hospital, Tianjin Medical University, Tianjin, 300060 China; 3 Tianjin Key Laboratory of Biomedical Detecting Techniques and Instruments, Tianjin, 300072 China

**Keywords:** Diffuse optical tomography, Inverse problem, Sparsity regularization, Non-negative

## Abstract

**Background:**

In diffuse optical tomography (DOT), the image reconstruction is often an ill-posed inverse problem, which is even more severe for breast DOT since there are considerably increasing unknowns to reconstruct with regard to the achievable number of measurements. One common way to address this ill-posedness is to introduce various regularization methods. There has been extensive research regarding constructing and optimizing objective functions. However, although these algorithms dramatically improved reconstruction images, few of them have designed an essentially differentiable objective function whose full gradient is easy to obtain to accelerate the optimization process.

**Methods:**

This paper introduces a new kind of non-negative prior information, designing differentiable objective functions for cases of L_1_-norm, L_p_ (0 < *p* < 1)-norm and L_0_-norm. Incorporating this non-negative prior information, it is easy to obtain the gradient of these differentiable objective functions, which is useful to guide the optimization process.

**Results:**

Performance analyses are conducted using both numerical and phantom experiments. In terms of spatial resolution, quantitativeness, gray resolution and execution time, the proposed methods perform better than the conventional regularization methods without this non-negative prior information.

**Conclusions:**

The proposed methods improves the reconstruction images using the introduced non-negative prior information. Furthermore, the non-negative constraint facilitates the gradient computation, accelerating the minimization of the objective functions.

## Background

Diffuse optical tomography (DOT) has attracted widespread interest in recent years due to its non-invasive [1, 2] and sensitive [3] properties, which offers huge clinical potential. This biomedical imaging modality presents lower radiation risk than those methods using X-ray [3] and has extensive applications including optical mammography and functional brain imaging [1, 4]. DOT reconstructs the spatial distribution of the optical properties such as absorption coefficients, which provide useful functional information about blood volume and oxygenation. However, the reconstructed images of DOT often suffer from the low spatial resolution and are significantly affected by measurement noise because the inverse problem of DOT is highly ill-posed [1–5]. The inverse problem of DOT is both undetermined because the available measurements are far fewer than the unknown variables to be reconstructed and ill-conditioned because of the dominance of scattering during the propagation of light in human tissues.

To alleviate this ill-posedness, researchers introduced various useful prior information to regularize the DOT inverse problem. The first introduced prior information is smoothness constrain incorporated in the well-established Tikhonov regularization method, which to some extent improved the reconstruction image. The involved optimization problem can be effectively solved because its objective function is differentiable. However, the reconstructed image of this method is often over-smoothed, due to the incorporated smoothness constraint, discouraging sharp edges in the reconstructed images. The second important prior information, used to alleviate the ill-posedness, is that the real solution is sparse. Its corresponding regularization term is based on the L_p_-norm (0 ≤ *p* ≤ 1) instead of L_2_-norm. These sparsity regularization methods can facilitate the recovery of the sharp edges and are robust with noise [4, 5]. This is based on the clinical fact that the breast tumor usually accounts for a small part of the overall breast, and the remaining part is healthy [2, 4]. Thus, the changes of the optical coefficients, which can be caused by the regional blood blow changes or the early stage of breast cancers are expected to be localized. That is to say, the original reconstruction problem itself is sparse. Up to now, researchers have devised a variety of sparsity regularization methods using different L_p_ (0 ≤ *p* ≤ 1)-norms [6, 7]. For example, in case of L_1_-norm, Amir Beck et al. proposed a practical method named FISTA [8] using the gradient information; Figueiredo et al. proposed a method called GPSR [9] to split the L_1_-norm into two parts, from which the gradient is easy to obtain. To use L_p_ (0 < *p* < 1)-norm regularization, several approaches [4, 10, 11] were proposed to obtain the optimal solution with the help of majorization-minimization framework. When *p* = 0, a well-established smooth L_0_-norm based regularization method [4, 12] was designed, which had proven to be effective. The total variation methods of special sparsity regularization [13] also improved the reconstruction image by using the L_1_-norm of the difference of neighboring pixels rather than that of the vector in the reconstructed image as the constraint term.

However, image reconstruction using these algorithms is a rather lengthy process. Their objective functions are essentially non-differentiable, unlike those of the Tikhonov method, making it impossible to use their gradient information directly to minimize them. More specifically, using these algorithms requires more computational work and time seeing as the full gradient information cannot be easily obtained to guide the optimization process.

In this paper, a new kind of non-negative prior information is introduced for the first time, designing regularization methods with differentiable objective functions for L_1_-norm, L_p_ (0 < *p* < 1)-norm and L_0_-norm. These regularization methods not only make full use of the gradient information of the objective functions, like the Tikhonov method, but also retain the advantages of the sparsity regularizations in improving image quality. To investigate the performances of these proposed methods, the methods with the non-negative constraint and those without the non-negative constraint are compared, using numerical simulation and phantom experiments.

## Methods

### The non-negative prior information

This work assumes that scattering coefficients are both known and spatially constant. Utilizing the finite element formulation to discretize the photon propagation model, the reconstruction of the absorption coefficients in DOT can be simplified into a linear equation with the following form:

1 $$\delta{\varvec{\Gamma}}= \bf{J}\delta{\varvec{\upmu}}_{a} + \bf{n},$$

where $\delta{\varvec{\Gamma}}\in {\bf{R}}^{{\rm{N}_{\rm{m}} }}$ is the measurement vector with the number of measurements $N_{m} \cdot \bf{J} \in {\bf{R}}^{N_{m} \times N_{n} }$ is the Jacobian matrix with the number of nodes *N*_*n*_ in the finite element method. $\delta{\varvec{\upmu}}_{\text{a}} \in {\mathbf{R}}^{{\rm{N}_{\rm{n}} }}$ refers to the perturbation of absorption coefficients. ${\bf{n}} \in {\bf{R}}^{{\rm{N}_{\rm{m}} }}$ is the additive noise introduced unavoidably during the measurement or the error in the computation.

The common regularization methods, using different prior information to solve the above linear equation, can be summarized with an optimization problem as follows

2 $$\delta {\varvec{\upmu}}_{a}^{*} = \arg \mathop {\hbox{min} }\limits_{{\delta {\varvec{\upmu}}_{a} }} \,\left\{ {\frac{1}{2}||{\mathbf{J}}\delta {\varvec{\upmu}}_{a} - \delta {\varvec{\Gamma}}||_{2}^{2} + \lambda | |\delta {\varvec{\upmu}}_{a} | |_{p}^{p} } \right\} ,$$

where the first term $\frac{1}{2}||{\mathbf{J}}\delta {\varvec{\upmu}}_{a} - \delta {\varvec{\Gamma}}||_{2}^{2}$ is the fidelity term and the second term $\lambda \left| {\left| {\delta {\varvec{\upmu}}_{a} } \right|} \right|_{{_{P} }}^{p}$ is the regularization term. The regularization parameter, *λ*, is a non-negative value balancing between the data fitting and L_p_-norm penalty (0 ≤ *p* ≤ 1). When p = 2, Eq. (2) is the well-known Tikhonov method. Equation (2) represents a variety of sparsity regularization methods, when 0 ≤ *p* ≤ 1. The fidelity term is differentiable but the regularization term is non-differentiable when 0 ≤ *p* ≤ 1, making it impossible to effectively minimize the objective function using the full gradient information.

To optimize Eq. (2) more efficiently and more precisely, a new kind of non-negative prior information is incorporated to constrain the solution. Specially speaking, from the viewpoint of physiology, the abnormal absorption coefficients caused by the breast tumor with angiogenesis must have bigger absorption coefficients than those of the normal region because the tumor has more hemoglobin [5]. Thus the changes of the absorption coefficients compared with the normal region are non-negative; that is to say, $\delta {\varvec{\upmu}}_{a} \ge 0$ can be used as an inequality constraint. This is the non-negative prior information, which will play important role in designing differentiable objective functions for sparsity regularization methods and reconstructing images of higher quality.

### L_1_-norm regularized reconstruction scheme with the non-negative constraint

When *p* = 1, the regularization term can be made differentiable by introducing the non-negative constraint. Specifically speaking, knowing $\delta {\varvec{\upmu}}_{\text{a}} \ge {\mathbf{0}}$ in advance, Eq. (2) can be simplified into the following simple problem

3 $$\delta {\varvec{\upmu}}_{a}^{*} = \arg \mathop {\hbox{min} }\limits_{{\delta {\varvec{\upmu}}_{a} \ge 0}} \left\{ {{\text{f}}(\delta {\varvec{\upmu}}_{a} ) = \frac{1}{2}||{\mathbf{J}}\delta {\varvec{\upmu}}_{a} - \delta {\varvec{\Gamma}}||_{2}^{2} + \lambda \sum\limits_{i = 1}^{{N_{n} }} {\delta {\varvec{\upmu}}_{ai} } } \right\} .$$

The objective function f(*δ* **μ**_*a*_) in (3) is differentiable, making it possible to optimize (3) using full one-order gradient information. The gradient of the objective function is ${\mathbf{g}} = {\mathbf{J}}^{T} *({\mathbf{J}}\delta {\varvec{\upmu}}_{\text{a}} - \delta {\varvec{\Gamma}}){ + }\uplambda {\mathbf{1}}_{\text{N}_{\text{n} }}$, where ${\mathbf{1}}_{\text{N}_{\text{n}}} = [1,1, \ldots ,1]^{T} \in {\mathbf{R}}^{{\text{N}_{\text{n}}} \times 1}$. The constraint $\delta {\varvec{\upmu}}_{\text{a}} \ge {\mathbf{0}}$ is a bound constraint, for which the gradient projection method [9] is particularly effective, further simplifying the optimization process. Before exhibiting in detail the full algorithm, the projection of any arbitrary vector $\delta {\varvec{\upmu}}_{a}$ onto the feasible region $[{\mathbf{L}},{\mathbf{U}}]$ is defined as follows: the *i*th component is given by

4 $${\text{P}}(\delta{\varvec{\upmu} _{a}},{\mathbf{L}},{\mathbf{U}})_{i} = {\text{ }}\left\{ {\begin{array}{*{20}ll} {{\mathbf{L}}_{i} } &\quad {{\text{if}}} \quad {\delta \mu _{{ai}} < {\mathbf{L}}_{i} } \\ {\delta {\mathbf{U}}_{{ai}} } &\quad {{\text{if}}} \quad {\delta \mu _{{ai}} \in [{\mathbf{L}}_{i} ,{\mathbf{U}}_{i} ]} \\ {{\mathbf{U}}_{i} {\text{ }}} &\quad {{\text{if}}} \quad {\delta \mu _{{ai}} > {\mathbf{U}}_{i} {\text{ }}} \\ \end{array} } \right..$$

The piecewise linear path $\delta {\varvec{\upmu}}_{a} (t)$ starting from the initial point and obtained by projecting the steepest descent direction at $\delta {\varvec{\upmu}}_{\text{a}}^{0}$ onto the feasible region is thus given by

5 $$\delta {\varvec{\upmu}}_{a} (t) = \text{P} (\delta {\varvec{\upmu}}_{{_{a} }}^{0} - t{\mathbf{g}},{\mathbf{L}},{\mathbf{U}}) ,$$

where ${\mathbf{g}} = \nabla \text{f} (\delta {\varvec{\upmu}}_{a}^{0} )$ and *t* is the stepsize. $\delta {\varvec{\upmu}}_{a} \ge {\mathbf{0}}$ is a bound constraint; that is to say, the upper bound is ${\mathbf{U}} = [\infty ,\infty , \ldots ,\infty ]^{\text{T}} \in {\mathbf{R}}^{\text{N}_{\text{n} \times 1}}$ and the lower bound is ${\mathbf{L}} = [0,0, \ldots ,0]^{\text{T}} \in {\mathbf{R}}^{\text{N}_{\text{n} \times 1}}$. This process is simple and requires less amount of computation, making it suitable for solving large-scale problems where the dimensions are very large.

The detailed gradient projection method for L_1_-norm regularized reconstruction scheme with non-negative constraint is shown below, according to the optimization theory. Hereinafter, this method will be referred to as the NL_1_ (Non-Negative L_1_) method for short.

### L_p_-norm regularized reconstruction scheme with the non-negative constraint

In the case of 0 < *p* < 1, the gradient of the regularization term is different from that of the L_1_-norm. Taking advantage of the non-negative prior information, the proposed objective function is

6 $$\delta {\varvec{\upmu}}_{a}^{*} = \arg \mathop {\hbox{min} }\limits_{{\delta {\varvec{\upmu}}_{a} \ge {\mathbf{0}}}} \left\{ {\text{f} (\delta {\varvec{\upmu}}_{a} ) = \frac{1}{2}||{\mathbf{J}} - \delta {\varvec{\Gamma}}||_{2}^{2} + \lambda \sum\limits_{i = 1}^{{N_{n} }} {\delta {\varvec{\upmu}}_{ai}^{p} } } \right\}$$

In this scenario, the gradient of the objective function is ${\mathbf{g}} = {\mathbf{J}}^{T} *({\mathbf{J}}\delta {\varvec{\upmu}}_{a} - \delta {\varvec{\Gamma}}){ + }\lambda \sum\nolimits_{i = 1}^{{N_{n} }} {\frac{1}{{\delta {\varvec{\upmu}}_{ai}^{1 - p} + C}}}$, where *C* is a small positive constant that provides stability by ensuring that the zero components of *δ* **μ**_a_ do not prohibit a nonzero estimate in the next step. Like the NL_1_ method algorithm, the NL_p_ (Non-Negative L_1_) algorithm is designed by replacing the gradient term with ${\mathbf{g}} = {\mathbf{J}}^{T} *({\mathbf{J}}\delta {\varvec{\upmu}}_{a} - \delta {\varvec{\Gamma}}){ + }\lambda \sum\nolimits_{i = 1}^{{N_{n} }} {\frac{1}{{\delta {\varvec{\upmu}}_{ai}^{1 - p} + C}}}$. The remaining part of this algorithm is the same as that of the NL_1_ algorithm. In this paper, the values of p and C are fixed at 1/2 and 1e-6, respectively.

### L_0_-norm regularized reconstruction scheme with the non-negative constraint

For *p* = 0, i.e., the NL_0_ (Non-Negative L_0_) method, the optimization problem is NP-hard, and cannot be solved efficiently. Incorporating the non-negative prior information and the approximate L_0_-norm, the proposed complete objective function is as follows

7 $$\delta {\varvec{\upmu}}_{a}^{*} = \arg \mathop {\hbox{min} }\limits_{{\delta {\varvec{\upmu}}_{a} \ge {\mathbf{0}}}} \left\{ {\text{f} (\delta {\varvec{\upmu}}_{a} ) = \frac{1}{2}||{\mathbf{J}} - \delta {\varvec{\Gamma}}||_{2}^{2} + \lambda \sum\limits_{i = 1}^{{N_{n} }} {\left[1 - e^{{ - \frac{{\delta {\varvec{\upmu}}_{ai}^{2} }}{{2\sigma^{2} }}}} \right]} } \right\} .$$

The parameter *σ* controls the degree of approximation. For smaller values of *σ*, the regularization term is more approximate to L_0_-norm and is more difficult to be minimized [12]. After ignoring constant terms, Eq. (7) can be rewritten as

8 $$\delta {\varvec{\upmu}}_{a}^{*} = \arg \mathop {\hbox{min} }\limits_{{\delta {\varvec{\upmu}}_{a} \ge {\mathbf{0}}}} \left\{ {f(\delta {\varvec{\upmu}}_{a} ) = \frac{1}{2}||{\mathbf{J}} - \delta {\varvec{\Gamma}}||_{2}^{2} + \lambda \sum\limits_{i = 1}^{{N_{n} }} {\left[ - e^{{ - \frac{{\delta {\varvec{\upmu}}_{ai}^{2} }}{{2\sigma^{2} }}}} \right]} } \right\} .$$

The introduction of the non-negative prior information makes it easy to calculate the gradient as

9 $${\mathbf{g}}\,=\,{\mathbf{J}}^{T} *({\mathbf{J}}\delta {\varvec{\upmu}}_{\text{a}} - \delta {\varvec{\Gamma}}) + \lambda \sum\limits_{i = 1}^{\text{N}_{\text{n} }} {\frac{{\delta {\varvec{\upmu}}_{ai} }}{{\upsigma^{2} }}e^{{ - \frac{{\delta {\varvec{\upmu}}_{ai}^{2} }}{{2\upsigma^{2} }}}} },$$

The whole algorithm for the NL_0_ (Non-Negative L_0_) is presented as follows using the pseudo code: 

## Numerical and phantom experiments

To evaluate the performances of the proposed regularization methods, several numerical and phantom experiments are conducted. Their reconstructed results are evaluated and then compared with those of the conventional regularization methods based on the same norm.

### Conditions

**Fig. 1:**
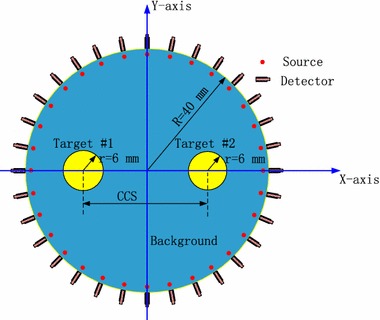
Structural schematic diagram of the medium. In both the numerical and phantom experiments for the spatial resolution evaluation, the CCS is set to 22, 26, and 30 mm, respectively. For the quantitativeness and gray resolution analysis, the CCS is fixed at 40 mm

As illustrated in Fig. 1, a 2-D circular breast-sized turbid medium with the radius of R = 40 mm is used for the investigations. Two circular tumor-emulating regions of 6-mm radius (Target #1 and Target #2) are embedded into the background medium symmetrical to the Y-axis, and have higher absorption coefficients. Depending on the purposes of the experiments, the center-to-center separation (*CCS*) between the two targets can be varied. In both the numerical and phantom experiments for the spatial resolution evaluation, the targets are located at (x = ±11 mm, y = 0 mm), (x = ±13 mm, y = 0 mm) and (x = ±15 mm, y = 0 mm), i.e., *CCS* = 22, 26 and 30 mm, respectively. In the experiments for the quantitativeness and gray resolution assessments, the targets are fixed at (x, y) = (x = ±20 mm, y = 0 mm), corresponding to *CCS* = 40 mm.

The optical properties of healthy and diseased breast tissues have been extensively investigated using both in vitro and in vivo methods [14]. Most investigations have measured the absorption and (reduced) scattering coefficients of healthy breast tissues in the range of 0.002–0.008 mm^−1^ and 0.6–1.3 mm^−1^, respectively, in the near-infrared wavelength range. The in vivo measurements reported by Tromberg et al. indicate that some tumors exhibit 1.25 to threefold higher absorption than normal breast tissue [15]. Overall, most evidence suggests that the ratio between the absorption for healthy and diseased tissues are of the order of a factor of 2 [14]. In this paper, to simulate the clinical cases, the absorption and (reduced) scattering coefficients of the background medium are set to 0.004 and 1 mm^−1^, respectively. For the target regions, the absorption coefficients vary with the purposes of the experiments while the scattering coefficients are the same as that of the background: for the spatial resolution, the absorption coefficient of the targets are set as 0.008 mm^−1^; for the quantitativeness, both the two targets have the same absorption coefficients of 0.005, 0.006 mm, and 0.008 mm^−1^, mimicking the tumors with contrasts of 1.25, 1.5 and 2.0, respectively. For the grayscale resolution, the two targets have different absorption coefficients, paired as (0.0072, 0.0088 mm^−1^), (0.0064, 0.0096 mm^−1^) and (0.0056, 0.0104 mm^−1^), corresponding to a grayscale difference of 10, 20 and 30%. It is worth noting that the same experimental setup, including the geometry and optical coefficients, are also used in the phantom experiments, facilitating the mutual collation between the numerical and phantom experiments.

Thirty-two source positions (red regions) evenly located one mean transport length inside the boundary are illuminated by the continuous-wave source one by one. For each illumination, except the 15 detectors nearest to the source position, the remaining 17 detectors evenly located on the surface of the medium are used to measure the intensities of the reemitted light, leading to a total of 32 × 17 measurements.

Four metrics are adopted to objectively select the optimal regularization parameters based on the reconstruction image quality [16]. First, the root mean square error (RMSE) is defined to measure the difference between the reconstructed and the true values

10 $$RMSE = \sqrt {\frac{{||\mu_{a} ({\mathbf{r}}) - \mu_{a}^{\text{tr}} ({\mathbf{r}})||_{2}^{2} }}{{||\mu_{a}^{\text{tr}} ({\mathbf{r}})||_{2}^{2} }}},$$

where *μ*_*a*_(**r**) and *μ*_*a*_^tr^(**r**) are the reconstructed and true absorption images at position **r**, respectively. Second, the area ratio (AR) is defined to measures the ratio of the reconstructed target area to the true one

11 $$AR = \frac{{|A^{\text{tg}} |}}{{|A^{\text{tr}} |}},$$

where *A* ^tr^ and *A* ^tg^ denotes the true and reconstructed target regions, respectively, with the latter defined as the voxels with their values higher than one half of the maximum of the reconstructed values, and|·| is the operator for area calculation. Third, the contrast-to-noise ratio (*CNR*) is defined to assess how the targets can be distinguished from the background

12 $$CNR = \frac{{\mathop {\text{Mean} }\nolimits_{{{\mathbf{r}} \in A^{tg} }} [{\varvec{\upmu}}_{a} ({\mathbf{r}})] - \mathop {\text{Mean}}\nolimits_{{{\mathbf{r}} \in A^{bg} }} [{\varvec{\upmu}}_{a} ({\mathbf{r}})]}}{{\sqrt {w\mathop {\text{Var}}\nolimits_{{{\mathbf{r}} \in A^{tg} }} [{\varvec{\upmu}}_{a} ({\mathbf{r}})] + (1 - w)\mathop {\text{Var}}\nolimits_{{{\mathbf{r}} \in A^{bg} }} [{\varvec{\upmu}}_{a} ({\mathbf{r}})]} }} ,$$

where *w* = |*A* ^tg^|/|*A* ^tg^|(|*A* ^tg^| + |*A* ^*bg*^|) with *A* ^*bg*^ representing the background region, ‘Mean’ and ‘Var’ operators for mean and variance calculations, respectively. Finally, to globally assess the reconstruction image quality, a total error (*TE*) is defined as follows:

13 $$TE = e^{{RMSE + absolute(AR)}} /CNR,$$

When optimizing the regularization parameter *λ*, *TE* is calculated as a function of the regularization parameter, *λ*. The *λ* producing the smallest *TE* is selected because it provides the best balance across the above 3 metrics.

For enforcing evaluation of the method performances, three further metrics, namely *SR, AC* and *GR*, are defined to quantitate the spatial resolution, quantitativeness and grayscale discerning ability of the reconstructed images, respectively [17]

14 $$SR = \frac{{\upmu_{a} (x)|_{x = 0} - \mathop {\hbox{min} }\nolimits_{x} [\upmu_{\text{a}} (x)]}}{{\mathop {\hbox{max} }\nolimits_{x} [\upmu_{a} (x)] - \mathop {\hbox{min} }\nolimits_{x} [\upmu_{a} (x)]}},$$

15 $$AC = \frac{{\mathop {\hbox{max} }\nolimits_{x} [\upmu_{a} (x)]}}{{\mathop {\hbox{min} }\nolimits_{x} [\upmu_{a} (x)]}},$$

16 $$GR = \frac{{\mathop {\hbox{max} }\nolimits_{x \ge 0} [\upmu_{a} (x)] - \mathop {\hbox{max} }\nolimits_{x \le 0} [\upmu_{a} (x)]}}{{\mathop {\hbox{max} }\nolimits_{x \ge 0} [\upmu_{a} (x)] + \mathop {\hbox{max} }\nolimits_{x \le 0} [\upmu_{a} (x)]}},$$

where μ_*a*_(*x*) is the profile of the absorption image along the X-axis, i.e., μ_*a*_(*x*) = μ_*a*_(**r**)|_**r**=(*x*,0)_.

Generally speaking, better image reconstruction can be identified by: smaller RMSE, TE, and SR values; a larger CNR value; an AR value closer to 1; and AC and GR values closer to the true values.

### Numerical experiments

To more closely simulate the real case, we assume that the main noises in the measurements are Gaussian noise [4–6]. Gaussian of different levels are added to the pure simulated measurements. The signal-to-noise ratio (*SNR*) of the detecting location with the weakest light is set to *SNR*_min_ = 20 and 30 dB, respectively. The best signal-to-noise ratio for a number of photons, N, reaching the detector in a given time interval is $SNR = \sqrt N$ [18]. So, the *SNR*s for the other detecting locations are set according to $SNR = SNR_{\rm{min} } \sqrt {I/I_{\rm{min} } }$, with *I* and *I*_min_ being the intensities of the current detecting locations and the one with the weakest light, respectively. Different meshes are adopted in the forward and inverse problems to avoid tricky problems. Specifically, a fine mesh with 12,290 nodes and 24,098 triangle elements and a coarse mesh with 4526 nodes and 8762 triangle elements are used for the forward and inverse calculations, respectively. To comprehensively analyze and compare the methods, we repeat each experiment 10 times and plot the average value and 95% confidence interval of the different metrics.

#### Spatial resolution

**Fig. 2:**
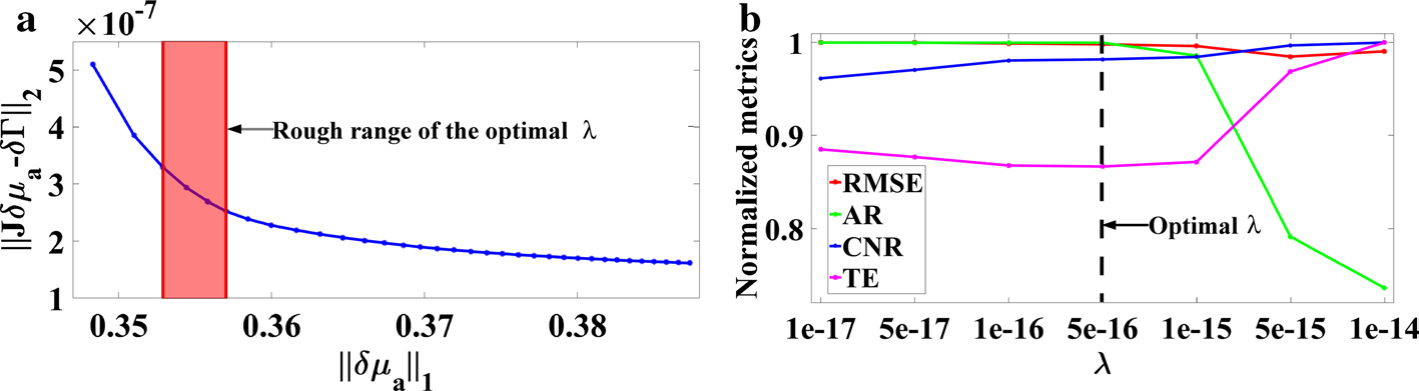
Selection of the optimal regularization parameter: **a** the generalized L-curve and the red rough range of the optimal *λ*; **b** comparison of the normalized metrics including *RMSE*, *AR*, *CNR* and *TE*. The optimal regularization parameter is *λ* = 5*e* − 16, as highlighted by the *dotted vertical line*

Before demonstrating the reconstructed results, we first illustrate an example about the process to select the optimal regularization parameter *λ* for the NL_*p*_ (*p* = 1/2) method in the spatial resolution experiments with a fixed *CCS* = 22 mm. This process consists of two steps. The first step is to find a rough range of optimal *λ* utilizing the generalized L-curve, shown in Fig. 2a. It traces, for a specific pair of ${\mathbf{J}}\,{\text{and}}\,\delta\varvec{\varGamma}$, the optimal tradeoff in the space covered by the L_2_-norm of residual and the L_p_-norm of the regularized solution. Like the L-curve of the Tikhonov regularization method, by locating the comer of the generalized L-curve, an approximation to the optimal regularization parameter can be obtained [19]. The corner is located in the range of [1e-17 1e-14] (The red region). In the second step, more regularization parameters in this rough range are investigated and their normalized metrics are plotted in Fig. 2b. From Fig. 2b, it is clear that AR decreases gradually as *λ* increases when *λ* ≤ 1*e* − 15, but decreases dramatically when *λ* ≥ 1*e* − 15. This indicates that too large a *λ* excessively localizes the targets so that the reconstructed targets are much smaller than the real ones. The RMSE decreases gradually in the whole range investigated, indicating that the difference between the reconstructed results and the true ones becomes smaller. The *CNR* increases as *λ* increases, meaning that the reconstructed targets are easier to be distinguished from the background. Most importantly, the TE, valuing the reconstruction error in total, first decreases slowly and then increases rapidly. In summary, *λ* = 5*e* − 16 corresponding to the smallest *TE*, is selected as the optimal regularization parameter. It is worth noting that the reconstruction is un-sensitive to the change of *λ* when *λ* ≤ 1*e* − 15. So, the method is robust with the regularization parameter *λ* smaller than 1e−15. For all the following experiments, this method to select the optimal regularization parameter is used.

**Fig. 3:**
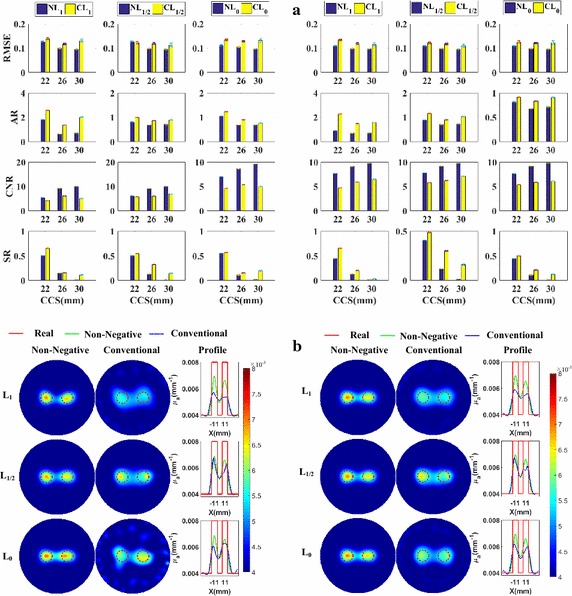
Numerical experiments for the spatial resolution analysis with absorption coefficients being 0.008 mm^−1^: **a** the evaluation metrics of *CCS* = 22, 26, and 30 mm, respectively, at *SNR*_min_ = 20 dB (*left*) and *SNR*_min_ = 30 dB (*right*); **b** the reconstructed images of *CCS* = 22 mm, at *SNR*_min_ = 20 dB (*left*) and *SNR*_min_ = 30 dB (*right*)

Figure 3a shows the column diagram of the metrics of the reconstructed images using different regularization methods. The first, second, and third columns present the metrics of the methods based on the L_1_-norm, L_1/2_-norm, and L_0_-norm respectively. Different rows contain different metrics labeled on the left side. The left and right parts contain the metrics for *SNR*_min_ = 20 dB and *SNR*_min_ = 30 dB, respectively. All the following figures presenting the metrics are likewise presented. Form Fig. 3a, we can observe that, excepting *AR*, all metrics of the non-negative methods are better than or comparable to those of the conventional methods based on the same norm. From the confidence intervals of the metrics, we can see that the non-negative methods are more robust with the noise.

Furthermore, to more clearly and directly demonstrate the results, we plot the reconstructed images for the cases of *CCS* = 22 mm in Fig. 3b. These are the most difficult cases to separate the two nearest targets and the best cases to highlight the differences of spatial resolution abilities between different methods. The first and second columns present the images reconstructed using the non-negative methods and the conventional methods without the non-negative constraint, respectively. The third column contains the profiles of the reconstructed absorption coefficients along the X-axis. The first, second, and third rows contain the results corresponding to the L_1_, L_1/2_ and L_0_-norm, respectively. To facilitate the comparison between the methods with and without the non-negative prior information, all the images are shown using the same colormap. The profiles for the same norm-based regularization methods are plotted in the same subfigures. The left and right parts contain the metrics for *SNR*_min_ = 20 dB and *SNR*_min_ = 30 dB, respectively. All the following figures presenting the images are likewise presented. From Fig. 3b, it is clear that the targets reconstructed using the proposed methods have smaller sizes and are better separated than those of the conventional methods based on the same norm. At the same time, the results of the non-negative regularization methods have less artifacts than those of the conventional methods. This validates the bigger *CNR* of the non-negative regularization methods compared to those of the conventional methods. This is because the proposed methods better suppress the influences of the noise and of the difference in the meshes used in the forward and inverse problems. Figure 3a, b jointly demonstrate that the non-negative regularization methods have better spatial resolution while retaining better *RMSE* and *CNR*.

#### Quantitativeness

**Fig. 4:**
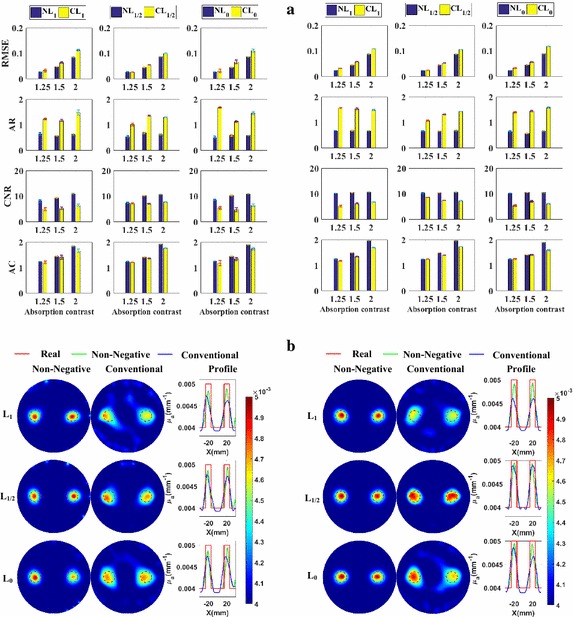
Numerical experiments for the quantitativeness analysis with *CCS* = 40 mm: **a** the evaluation metrics with absorption coefficients being 0.005, 0.006, and 0.008 mm^−1^, respectively, at *SNR*_min_ = 20 dB (*left*) and *SNR*_min_ = 30 dB (*right*); **b** the reconstructed images with absorption coefficients being 0.005 mm^−1^, at *SNR*_min_ = 20 dB (*left*) and *SNR*_min_ = 30 dB (*right*)

To focus on studying the quantitativeness of the methods, the *CCS* is fixed at 40 mm, which is far enough for the methods to separate the two targets. The metrics of the reconstructed images using different regularization methods are shown in Fig. 4a. Excepting the *AR*, all metrics of the non-negative methods are better than those of the conventional methods based on the same norm.

Furthermore, to more clearly and directly demonstrate the results, we plot the reconstructed images for the cases of *AC* = 1.25 in Fig. 4b. These are the most difficult cases to distinguish the targets from the background, due to the low contrast, and the best cases to highlight the difference of quantitativeness between different methods. From Fig. 4b, we observe that the images of the non-negative regularization methods have less artifacts and are better distinguished from the background than those of the conventional methods based on the same norm. Figure 4a, b jointly demonstrate that the non-negative regularization methods have better quantitativeness abilities while retaining better *RMSE* and *CNR*.

#### Grayscale resolution

**Fig. 5:**
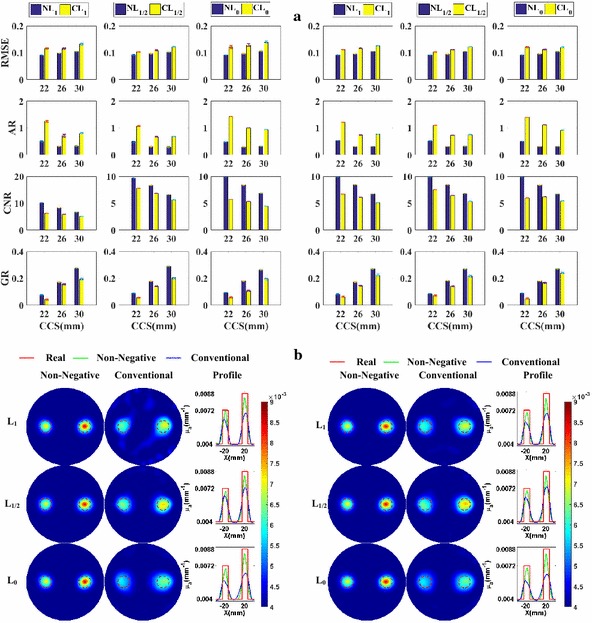
Numerical experiments for the grayscale resolution analysis of *CCS* = 40 mm: **a** the metrics with absorption coefficients paired as (0.0072, 0.0088 mm^−1^), (0.0064, 0.0096 mm^−1^), and (0.0056, 0.0104 mm^−1^), respectively, at *SNR*_min_ = 20 dB (*left*) and *SNR*_min_ = 30 dB (*right*); **b** The reconstructed images of absorption coefficients paired as (0.0072, 0.0088 mm^−1^), at *SNR*_min_ = 20 dB (*left*) and *SNR*_min_ = 30 dB (*right*)

The metrics of the reconstructed images using different regularization methods are plotted in Fig. 5a. Figure 5a shows that all the metrics, except the *AR*, of the reconstructed images of the non-negative methods are better than those of their conventional methods based on the same norm.

Furthermore, to more clearly and directly demonstrate the results, we plot the reconstructed images for the cases with a *GR* being 10% in Fig. 5b. These are the most difficult cases to reconstruct the small grayscale difference, and the best cases to highlight the difference of grayscale resolution between different methods. It is easy to find that targets reconstructed using the non-negative methods have less artifacts and closer grayscale difference to the real values. Figure 5a, b jointly demonstrate that the non-negative regularization methods have better grayscale resolution ability while retaining better *RMSE* and *CNR*.

#### Execution time

**Fig. 6:**
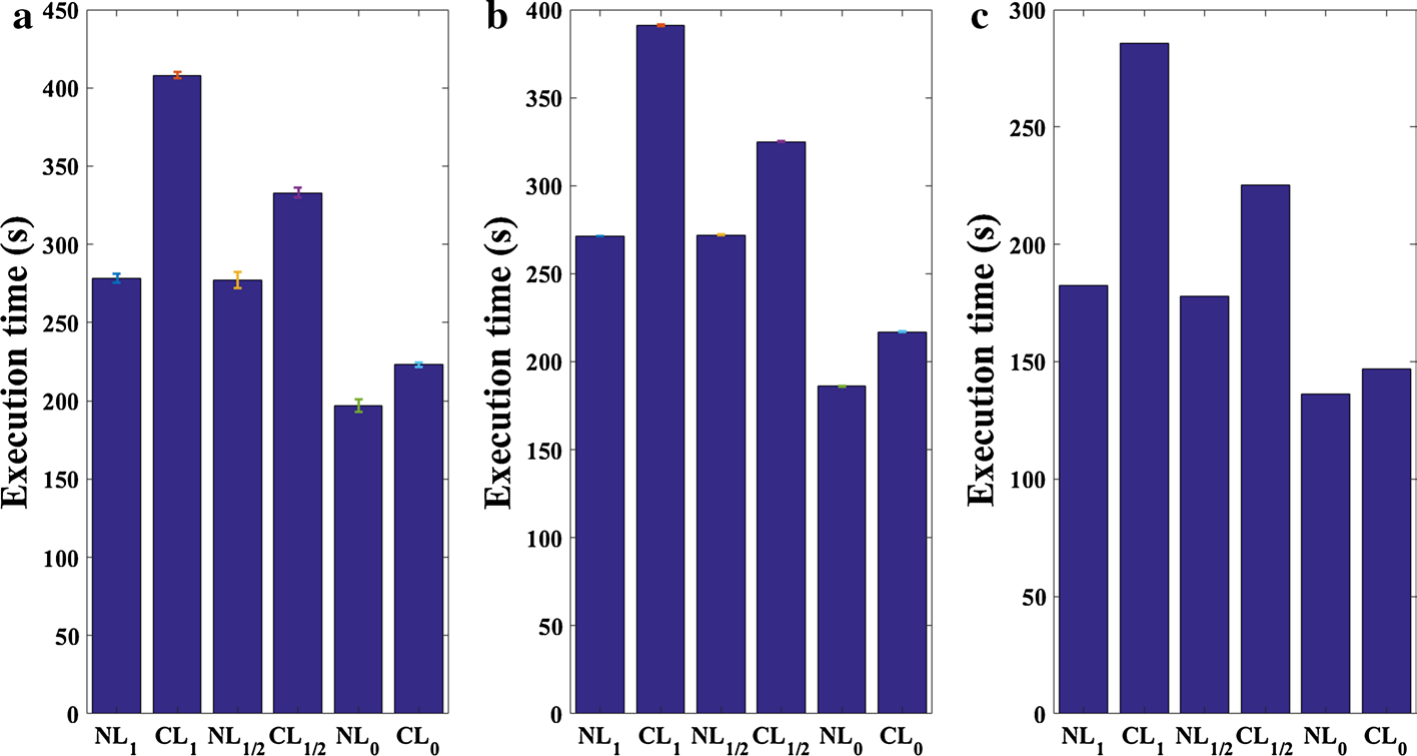
Comparisons of the execution time required by different regularization methods for the spatial resolution analysis with CCS = 22 mm: **a** the numerical experiments with *SNR*_min_ = 20 dB; **b** the numerical experiments with *SNR*_min_ = 30 dB; **c** the phantom experiments

To compare the speed of the proposed methods, all the involved methods are investigated in the same numerical experiments. During the previous numerical experiments, analyzing the spatial resolution for the case of *CCS* = 20 mm, the execution time for all methods are recorded. In these experiments, the stopping criteria are the same, as shown in the algorithms of NL_1_, NL_1/2_ and NL_0_. The same computer, Interl(R) Core(TM) i7-4790 CPU @3.60 GHz, is used in all these experiments. The execution time averaged over 10 repeated experiments and the 95% confidence interval are listed in Fig. 6a, b. From Fig. 6a, b, we can see that the non-negative regularization methods not only obtain images with better quality but also require shorter time than the conventional methods based on the same norm.

### Phantom experiment

**Fig. 7:**
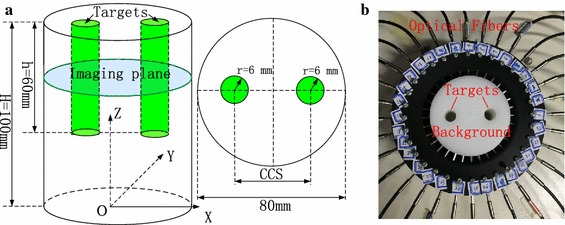
The phantom and optode arrangement: (**a**)The sketch of the phantom; (**b**) The phantom photo and optode arrangement

To further investigate the methods proposed in this paper, a continuous wave system [20] is used to conduct phantom experiments. A cylindrical polyoxymethylene phantom with the same cross section as that of the numerical experiments is adopted. The cross section of this phantom is discretized into a mesh with 4526 nodes and 8762 triangle elements, identical to that of the numerical scenario. The detailed structure of this phantom is shown in Fig. 7a. The phantom photo and optode arrangement are shown in Fig. 7b. Via the time domain system [21], the absorption and (reduced) scattering coefficients of this phantom at a wavelength of 675 nm are measured as 0.004 and 1 mm^−1^, identical to those of the background regions in numerical simulations. As described in the *Condition* section, the same experiments as those of the numerical experiments are conducted for the following phantom experiments. It is worth noting that we just conduct these phantom experiments once time and do not consider the experiments with different levels of noises.

Along the boundary of cylindrical phantom, 32 optodes with a coaxial structure of source and detector are placed equidistantly from one another (as shown in Fig. 7b). A steady state laser operating at a wavelength of 675 nm stimulates the phantom boundary through a 32:1 mechanical optical switch. Meanwhile, the reemitted light on the phantom boundary is collected and sent to 4 photomultiplier tubes (PMT) through four 8:1 mechanical optical switches. Then the single electron responses from the PMTs are detected and accumulated by the field programmable gate array (FPGA). The total number accumulated in 250 ms is used as the intensity of the light on the detecting locations on the phantom boundary. It is worth noting that the light of the locations near the sources is so strong that it possibly induces the stack effect, making the measurement imprecise. Therefore, only the remaining opposite 17 measurements farthest away from the sources are used. A total of 32 × 17 = 544 measurements, equal to that in the numerical experiments, are then used to reconstruct the spatial distribution of absorption coefficients.

All phantom experiments are conducted according to the following process. First, the holes embedded in the phantom are filled with the mixed solution having absorption and (reduced) scattering coefficients of 0.004 and 1 mm^−1^, identical to those of the healthy breast tissue. One of the optical fibers works as a light source and the remaining opposite 17 optical fibers are used to measure the light intensity reemitted from the surface of the phantom. Then, the remaining optical fibers work as the light source one by one, and the corresponding light intensities are measured. The mixed solution in the target holes is then replaced with the mixed solution with larger absorption coefficients, mimicking the breast tumors of different absorption contrasts. Repeating the measurement process, another 544 measurements are obtained and then are utilized in the reconstruction process.

#### Spatial resolution analysis

**Fig. 8:**
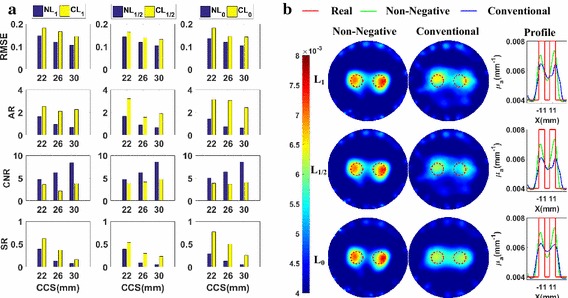
The phantom experiments for the spatial resolution analysis with absorption coefficients being 0.008 mm^−1^: **a** their evaluation metrics with CCS = 22, 26, and 30 mm, respectively; **b** the reconstructed images with CCS = 22 mm

The metrics of the reconstructed images are plotted in Fig. 8a. This figure shows that, excepting the *AR*, the metrics of the reconstructed images of the non-negative regularization methods are better than those of the conventional methods without the non-negative constraint based on the same norm.

Furthermore, to more clearly and directly demonstrate the results, we plot the reconstructed images for the cases of *CCS* = 22 mm in Fig. 8b. These are the most difficult cases to separate the two nearest targets and the best cases to highlight the differences of spatial resolution abilities between different methods. Figure 8b tells us that the images of the non-negative regularization methods have less artifacts and the two targets are better separated than those of the conventional methods. Figure 8a, b jointly demonstrate that the non-negative regularization methods have better spatial resolution ability while retaining better *RMSE* and *CNR*.

#### Quantitativeness

**Fig. 9:**
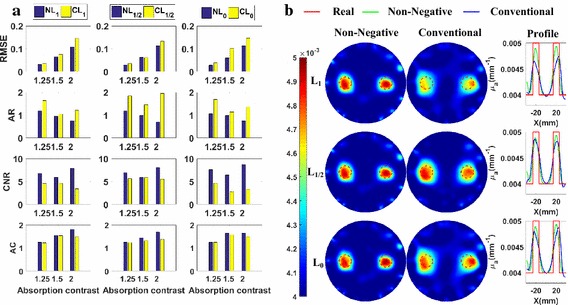
The phantom experiments for the quantitativeness analysis with *CCS* = 40 mm: **a** the metrics with absorption coefficients being 0.005, 0.006, and 0.008 mm^−1^, respectively; **b** The reconstructed images with absorption coefficients being 0.005 mm^−1^

We plot the metrics of the images reconstructed using the different methods in Fig. 9a. It is clear that, the metrics, except the *AR*, of the reconstructed images of non-negative methods are better than those of the conventional methods based on the same norm.

Furthermore, to more clearly and directly demonstrate the results, we plot the reconstructed images for the cases of *AC* = 1.25 in Fig. 9b. These are the most difficult cases to distinguish the targets from the background, due to the low contrast, and the best cases to highlight the difference of quantitativeness between different methods. From Fig. 9b, we observe that the images of the non-negative regularization methods have less artifacts and are better distinguished from the background than those of the conventional methods. Figure 9a, b jointly demonstrate that the non-negative regularization methods have better quantitativeness ability while retaining better *RMSE* and *CNR*.

#### Grayscale resolution

**Fig. 10:**
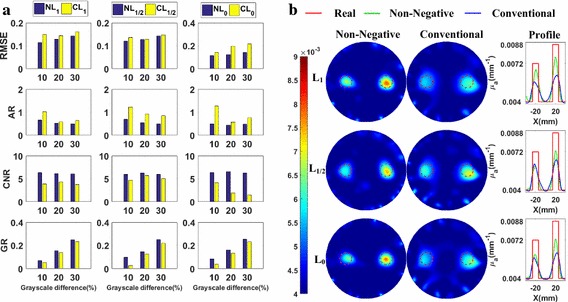
Phantom experiments for the grayscale resolution analysis with CCS = 40 mm: **a** the metrics with absorption coefficients paired as (0.0072, 0.0088 mm^−1^), (0.0064, 0.0096 mm^−1^), and (0.0056, 0.0104 mm^−1^), respectively; **b** the reconstructed images with absorption coefficients paired as (0.0072, 0.0088 mm^−1^)

Figure 10a contains the metrics of the images reconstructed using the different regularization methods. From this figure, it is clear that the metrics, except the *AR*, of the non-negative regularization methods are better than those of the conventional regularization methods based on the same norm.

Furthermore, to more clearly and directly demonstrate the results, we present the reconstructed images for the cases of a grayscale difference of 10% in Fig. 10b. These are the most difficult cases to reconstruct the small grayscale difference, and the best cases to highlight the difference of grayscale resolution between different methods. Figure 10b shows that the targets reconstructed using the non-negative methods have less artifacts and closer grayscale difference to the real values than those of the conventional methods. Figure 10a, b jointly demonstrate that the non-negative regularization methods have better grayscale resolution abilities while retaining better *RMSE* and *CNR*.

#### Execution time

The same stopping criteria and computer as those of the numerical simulation experiments are also adopted. Execution time of all the regularization methods for the phantom experiments analyzing spatial resolution for *CCS* = 22 mm are recorded and presented in Fig. 6c. It is obvious that the non-negative regularization methods require less time to obtain the optical solution than those of the conventional regularization methods based on the same norm.

## Discussion and conclusion

Though the investigations described in this paper are implemented on the continuous wave breast diffuse optical tomography, they can easily be extended to diffuse fluorescence tomography, because they share the same sparse and non-negative prior information.

The gradient projection methods described above can be replaced with a logarithmic barrier method. For the L_1_-norm, the involved unconstrained optimization problem is as follows:

17 $$\delta {\varvec{\upmu}}_{a}^{*} = \text{argmin} \left\{ {P(\delta {\varvec{\upmu}}_{a} ;\xi ) = \frac{1}{2}||{\mathbf{J}}\delta {\varvec{\upmu}}_{a} - \delta {\varvec{\Gamma}}||_{2}^{2} + \lambda \sum\limits_{i = 1}^{{N_{n} }} {\delta {\varvec{\upmu}}_{ai} } - \frac{1}{\xi }\log(\delta {\varvec{\upmu}}_{a} )} \right\}$$

where *ξ* is referred to as the barrier parameter. The gradient of the objective function is ${\mathbf{J}}^{T} ({\mathbf{J}}\delta {\varvec{\upmu}}_{a} - \delta {\varvec{\Gamma}}) + \lambda {\mathbf{1}}_{{N_{n} }} - \frac{1}{\xi }\frac{1}{{\delta {\varvec{\upmu}}_{a} + C}}$, which is easy to compute. However, it requires too much time to find an appropriate barrier parameter *ξ*, making it unpractical. This logarithmic barrier method also applies to the non-negative L_p_-norm (0 < p < 1) and L_0_-norm based regularization methods, but they also require much time to find the suitable barrier parameter.

In conclusion, we propose several new regularized methods based on L_1_-norm, L_p_-norm (0 < p < 1) and approximate L_0_-norm with non-negative prior information. Both numerical and phantom experiments demonstrate that, excepting *AR*, all metrics are improved by these proposed methods. Furthermore, the proposed methods require less time than those of the conventional methods based on the same norm.

## Abbreviations

DOT: diffuse optical tomography
CCS: center-to-center separation
RMSE: root mean square error
AR: area ratio
CNR: contrast-to-noise ratio
TE: total error
SR: spatial resolution
AC: absorption contrast
GR: grayscale resolution
*SNR*_min_: the minimum signal noise ratio
PMT: photomultiplier tubes
FPGA: field programmable gate array
Non-Neg-L_1_: L_1_-norm regularized reconstruction scheme with non-negative constraint
